# The Chemical Man

**Published:** 1887-04

**Authors:** 


The Chemical Man.—A man weighing 70 kilograms (156 lbs.) is composed of 13 elements, 5 gaseous and 8 solid. But man is principally composed of compressed oxygen, 44 kilograms, which at ordinary temperatures would occupy a space of 30 cubic metres. The quantity of hydrogen is less, 7 kilograms, which in a free state would occupy a space of 80 cubic metres, and the hydrogen of 12 men would fill a baloon capable of carrying 3 or 4 persons. The amount of nitrogen is 1 kilogram and 72 grams; Chlorine, 800 grams; fluorine, 100 grams. Of the solid elements of the body carbon is the most abundant, 22 kilograms. Besides this there are 800 grams of phosphorus and 100 grams of sulphur. There are no precious metals in man, but there are 1750 grams of calcium, 80 grams of potassium, 70 grams of sodium, 50 of magnesium and 50 of iron. Such is man.—*L' Sperimentale.*

